# Neuroprotective Effect of the Endogenous Amine 1MeTIQ in an Animal Model of Parkinson’s Disease

**DOI:** 10.1007/s12640-015-9556-6

**Published:** 2015-08-25

**Authors:** Agnieszka Wąsik, Irena Romańska, Jerzy Michaluk, Agnieszka Zelek-Molik, Irena Nalepa, Lucyna Antkiewicz-Michaluk

**Affiliations:** Department of Neurochemistry, Institute of Pharmacology Polish Academy of Sciences, 12 Smetna Street, 31-343 Krakow, Poland; Department of Biochemistry, Institute of Pharmacology Polish Academy of Sciences, 12 Smetna Street, 31-343 Krakow, Poland

**Keywords:** 1-methyl-12,3,4-tetrahydroisoquinoline, 1-benzyl-1,2,3,4-tetrahydroisoquinoline, Parkinson’s disease, Dopamine metabolism

## Abstract

Parkinson’s disease (PD) is a neurodegenerative disorder that is hallmarked by pathological changes associated with the death of dopaminergic neurons, particularly in the extrapyramidal system (substantia nigra pars compacta, striatum) of the brain. Although the causes of slow neuronal death in PD are unknown, both genetic and environmental factors are likely involved. Endogenous isoquinolines, such as 1-benzyl-1,2,3,4-tetrahydroisoquinoline (1BnTIQ), present in the human brain have been previously reported to participate in the pathogenesis of PD. The chronic administration of 1BnTIQ induced parkinsonism in primates, and this effect might be associated with idiopathic PD. However, another endogenous derivative of tetrahydroisoquinoline, 1-methyl-1,2,3,4-tetrahydroisoquinoline (1MeTIQ), displays clear neuroprotective properties in the brain. In the present study, we investigated the neuroprotective effects of 1MeTIQ (25 and 50 mg/kg) in an animal model of PD after the chronic administration of 1BnTIQ (25 mg/kg). Behavioral analyses demonstrate that both acute and repeated treatment with 1MeTIQ completely antagonized 1BnTIQ-induced changes in rat locomotor activity. Neurochemical experiments indicate that 1MeTIQ co-administered with 1BnTIQ completely antagonized 1BnTIQ-induced reduction in the dopamine (DA) concentration in rat brain structures. In conclusion, the results demonstrate that 1MeTIQ possesses important neuroprotective properties in the animal model of PD and that the rats did not develop tolerance after its chronic administration.

## Introduction

Tetrahydroisoquinolines, e.g., salsolinol and 1-benzyl-1,2,3,4-tetrahydroisoquinoline (1BnTIQ), are a group of endogenous compounds with neurotoxic properties. However, one derivative from this group, 1-methyl-1,2,3,4-tetrahydroisoquinoline (1MeTIQ), exhibits neuroprotective properties. It has previously been shown that 1MeTIQ acts as a reversible inhibitor of monoamino oxidase (MAO) activity, blocks both the formation of 3,4-dihydroxyphenylacetic acid (DOPAC) and the production of free radicals and shifts dopamine (DA) catabolism toward COMT-dependent *O*-methylation. This mechanism might be important for the neuroprotective activity of 1MeTIQ (Antkiewicz-Michaluk et al. 2001; Patsenka and Antkiewicz-Michaluk 2004). Additionally, 1MeTIQ acts as a natural scavenger of free radicals and interacts with the agonistic conformation of dopamine receptors (Antkiewicz-Michaluk et al. 2006, 2007). However, another endogenous amine present in the brain, 1BnTIQ, exhibits a contrasting molecular mechanism because this molecule enhances the rate of dopamine metabolism, activates the dopamine oxidation pathway, and increases the production of free radicals. In addition, 1BnTIQ inhibits the COMT-dependent *O*-methylation pathway (Antkiewicz-Michaluk et al. 2001; Wąsik et al. 2009). Reflecting the neurotoxic mechanism of 1BnTIQ, this compound is considered an etiological factor of idiopathic Parkinson’s disease (PD) (Kotake et al. 1995). Previous studies have demonstrated that chronic treatment with 1BnTIQ produces parkinsonian-like symptoms in rodents and primates (Kotake et al. 1995; 2003; Kohta et al. 2010). In vitro studies indicated that 1BnTIQ induces cell death via apoptosis and produces an increase in the formation of the active caspase-3 protein fragments (Shavali and Ebadi 2003). 1BnTIQ accumulates in the dopaminergic neurons, where this molecule might exert pathological effects that lead to parkinsonism. Kotake et al. (1995) showed that the level of 1BnTIQ in the cerebrospinal fluid (CSF) of parkinsonian patients was three times higher than that in the CSF of the control group. These data suggest that chronic treatment with a low dose of 1BnTIQ might serve as an adequate animal model of the progressive process of PD.

The aim of the present study was to investigate the neuroprotective effects of 1MeTIQ in an animal model of PD after the chronic administration of 1BnTIQ. Using behavioral tests, we analyzed the influence of acute and chronic treatment with 1MeTIQ on changes in rat locomotor activity induced through the repeated administration of 1BnTIQ.

In neurochemical experiments, using high-performance liquid chromatography (HPLC) methodology with electrochemical detection, we investigated the effect of 1MeTIQ on disturbances in brain DA metabolism evoked through 1BnTIQ. Additionally, the concentration of excitatory amino acids (EAA) in the frontal cortex and the level of α-synuclein and tyrosine hydroxylase (TH) in the substantia nigra were also detected.

## Materials and Methods

### Animals and Treatments

All experiments were performed in male Wistar rats with an initial body weight of 220–240 g. All animals had free access to standard laboratory food and tap water and were kept at room temperature (22°C) under an artificial light/dark cycle (12/12 h, light on at 7:00).

1BnTIQ was intraperitoneally (i.p.) administered at a dose of 25 mg/kg chronically for 14 consecutive days. In the mixed group, a single or chronic dose of 1MeTIQ (25 or 50 mg/kg i.p.) was administered 20 min before 1BnTIQ administration. Control rats were treated with the appropriate vehicle. Rats were sacrificed through decapitation at 2 h after the last drug injections, and different brain structures were dissected for further analysis. The experiments were performed between 9:00 and 16:00.

All experimental procedures were performed in accordance with the National Institutes of Health Guide for the Care and Use of Laboratory Animals and approved through the Bioethics Commission as compliant with Polish Law. All experimental procedures were approved through the Local Bioethics Commission of the Institute of Pharmacology, Polish Academy of Sciences in Kraków.

### Drugs

1MeTIQ hydrochloride and 1BnTIQ hydrochloride were synthesized (according to Cannon and Webster 1958) at the Department of Drug Chemistry of the Institute of Pharmacology, the Polish Academy of Sciences in Krakow. The purity of the compound was verified by measuring the melting point, and homogeneity was assessed using a chromatographic column. The compounds were dissolved in a 0.9 % NaCl solution.

### Behavioral Study

#### Locomotor Activity

Locomotor activity was assessed using actometers (Opto-Varimex activity monitors; Columbus Inst., USA) linked on-line to a compatible IBM PC. Each cage (43 × 44 × 25 cm) perimeter was lined with an array of 15 × 15 photocell beams located 3 cm from the floor surface. Interruptions of the photocell beams were counted as a measure of horizontal locomotor activity and defined as the distance traveled (in cm). The rats were administered an acute dose of 25 or 50 mg/kg (i.p.) 1MeTIQ (expression treatment group) or as the last dose (14-day chronic administration; development treatment group) at 20 min before the last dose of 1BnTIQ (25 mg/kg i.p.; 14-day chronic administration). Subsequently, the animals were transferred to experimental cages, and locomotor activity (horizontal activity, traveled distance in cm) and rearing (vertical activity, time in sec) were recorded for 90 min and analyzed using the Auto-Track Software Program (Columbus Instruments, USA). Each group comprised seven animals.

### Biochemical Studies

#### Ex Vivo Experiments

##### Dopamine Metabolism

The rats were sacrificed through decapitation 2 h after the last 1BnTIQ injection, and the nucleus accumbens and striatum were immediately dissected. The tissue was frozen on dry ice (−70  C) until further use in a biochemical assay. The levels of dopamine (DA) and its metabolites, 3,4-dihydroxyphenylacetic acid (DOPAC), 3-methoxytyramine (3-MT), and homovanillic acid (HVA), were assayed through high-performance liquid chromatography (HPLC) with electrochemical detection (Hewlett Packard 1049A). The tissue samples were weighed and homogenized in ice-cold 0.1 M perchloroacetic acid containing 0.05 mM ascorbic acid. After centrifugation (10,000*g* for 5 min), the supernatants were filtered through RC 58 0.2-im cellulose membranes (Bioanalytical Systems, West Lafayette, IN, USA). The HP 1050 chromatograph (Hewlett-Packard, Golden, CO, USA) was equipped with C18 columns. The electrochemical cell potential was 800 mV. The mobile phase comprised 0.05 M citrate–phosphate buffer (pH 3.5), 0.1 mM EDTA, 1 mM sodium octyl sulfonate, and 3.5 % methanol. The flow rate was maintained at 1 ml/min. Dopamine and its metabolites were quantified based on the chromatograph peak height compared with standards run on the day of analysis. Each group comprised seven animals.

##### Determination of Amino Acid Neurotransmitters

The rats were sacrificed through decapitation 2 h after the last 1BnTIQ injection, and the frontal cortex was immediately dissected. The tissue was frozen on dry ice (−70 °C) until further use in a biochemical assay. The tissue was weighed, and subsequently homogenized in 4 ml of chilled saline solution (0.9 %). The homogenate was centrifuged at 22.000*g* for 10 min at 4 °C. The supernatant was transferred and filtered through a 0.22-µm pore size Millipore filter for derivatization. A 100-µl aliquot of mixed amino acid solution or sample supernatant, 175 µl of borate buffer solution, 200 µl of acetonitrile, and 25 µl NBD-F working solution were mixed in a 1.5 ml centrifuge tube. The well mixed solution was incubated in a 60 °C water bath for 7 min in the dark. NBD-F reacts with amino group and enables temperature, 10 µl of solution was injected into the equilibrated HPLC system. The analysis was performed on a Coulochem III HPLC system equipped with a UV DIONEX UltiMate 3000 detector (472 nm). The analyte was separated on an analytical Hypersil C18 column (250 mm length × 4.6 mm, 5 µm particle diameter) manufactured in Germany. The mobile phase comprised a mixture of acetonitrile–phosphate buffer (0.02 mol/l, pH 6.0; 16:84, v/v) filtered through a 0.45-µm nylon filter and degassed under ultrasound and vacuum for 30 min. The mobile phase was delivered at a flow rate of 1.0 ml/min, and the column temperature was set to 30 °C.

##### Immunoblotting

Protein was extracted through high-speed shaking in plastic tubes with stainless steel beads in a tissuelyser with 100 μl of ice-cold RIPA lysis buffer (Sigma, USA), containing complete mini protease inhibitor (Roche Diagnostics, USA). After incubation for 30 min, the homogenates were centrifuged at 10,000*g* for 20 min at 4 °C.The resulting supernatants were collected and subjected to protein analysis using the Bicinchoninic Acid Assay Kit (Sigma, USA). Equal amounts of protein extracts (12 µg) were boiled in Laemmli buffer containing 5 % β-mercaptoethanol for 5 min, separated through SDS-PAGE (4–15 %), and transferred to nitrocellulose membranes. The membranes were blocked with 5 % non-fat dry milk in Tris-buffered saline containing Tween-20 (TBST; pH = 7.6) for 1 h at room temperature and incubated with a primary antibody against tyrosine hydroxylase (1:2,000; Cell Signaling, USA) or α-synuclein (1:2,000; Cell Signaling, USA) overnight at 4 °C. After three washes with blocking solution, the membranes were incubated with the appropriate secondary antibodies for 1 h at room temperature, followed by three washes with TBST. Antibody binding was detected using an enhanced chemiluminescence kit (ECL Plus, Pierce, USA). Equal loading proteins were further confirmed after probing with anti-calnexin antiserum (CNX; 1:5,000; Enzo Life Sciences, USA) or anti-β-actin antiserum (1:5,000; Sigma, USA). All Western blot analyses were performed at least twice to confirm the results. The chemiluminescence signal was visualized using a Luminescent Image Analyzer Fuji-Las 4000 (Fuji, Japan). Immunoreactive bands were quantified using an image analyzer (ScienceLab, MultiGauge V3.0).

### Calculations and Statistics

Two-way analysis of variance (ANOVA) for repeated measures was used to analyze the results of the behavioral test (locomotor activity). Differences between the control and experimental groups were assessed using Duncan’s post hoc test.

The comparison of the effects of acute and multiple administration of 1BnTIQ and the effect of chronic 1MeTIQ administration on 1BnTIQ-produced changes in the concentration of EAA were analyzed by one-way ANOVA, followed by Duncan’s post hoc test. The results of the biochemical experiments were analyzed using two-way ANOVA, followed by Duncan’s post hoc test, when appropriate. These indices were calculated using the concentrations of individual tissue samples (*n* = 6–7).

## Results

### Behavioral Study

#### The Effect of Acute 1MeTIQ Administration on 1BnTIQ-Induced Hyperactivity in Rats

**Fig. 1:**
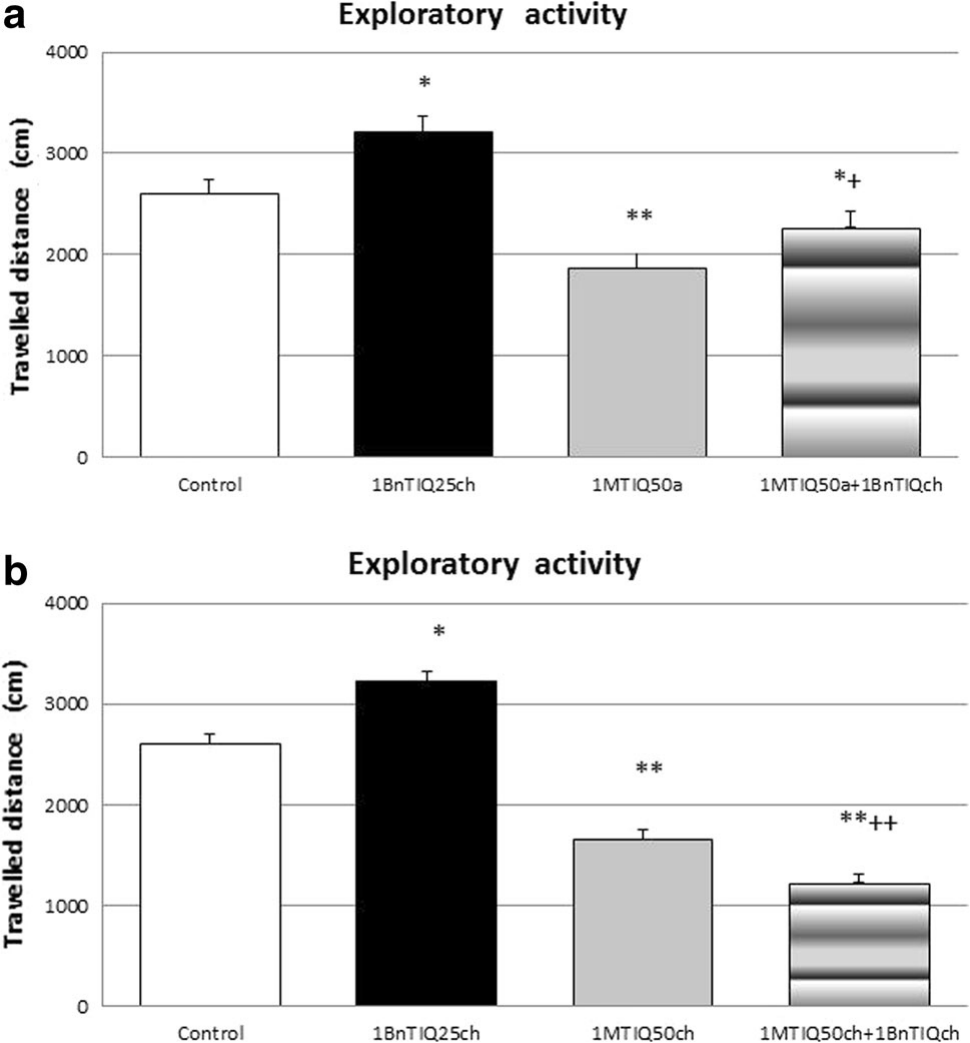
The influence of acute (**a**) and chronic (**b**) treatment with 1MeTIQ on changes in exploratory activity induced by chronic administration of 1BnTIQ. 1BnTIQ (25 mg/kg i.p.) was administered chronic during 14 consecutive days. 1MeTIQ (50 mg/kg i.p) was given acute (**a**) or chronic during 14 consecutive days (**b**). In the mixed group, 1MeTIQ was injected 20 min before 1BnTIQ administration. A control group was treated by saline. Rats were placed into actometers immediately after last drugs administration. Movements were recorded for 30 min. The data are expressed as the means ± SEM (*n* = 7 animals). Data were analyzed with a two-way ANOVA for repeated measures, followed by Duncan’s post hoc test. Statistical significance: * *P* < 0.05; ** *P* < 0.01 versus saline-treated group; ^+^ *P* < 0.05 versus 1BnTIQ-treated group

The acute administration of 1MeTIQ (50 mg/kg i.p.) induced a significant reduction in the horizontal exploratory activity of rats (*P* < 0.01). In addition, the chronic administration of 1BnTIQ (25 mg/kg i.p.) produced a significant (*P* < 0.05) elevation of exploratory activity (Fig. 1a). In the mixed group, an acute dose of 1MeTIQ completely antagonized 1BnTIQ-induced hyperactivity.

#### The Effect of Chronic 1MeTIQ Administration on 1BnTIQ-Induced Hyperactivity in Rats

As shown in Fig. 1b, the chronic administration of 1MeTIQ (50 mg/kg i.p.) over 14 days induced a significant (*P* < 0.01) decrease in exploratory activity. The multiple (14-day) administration of 1BnTIQ at a low dose of 25 mg/kg (i.p.) significantly increased (*P* < 0.05) the exploratory activity during the first 30 min of measurement. In the mixed group, 1MeTIQ completely antagonized 1BnTIQ-induced hyperactivity (Fig. 1b).

### Biochemical Studies

#### Ex Vivo Experiments

##### The Comparison of the Effects of the Acute and Multiple Administration of 1BnTIQ on Dopamine Metabolism in Rat Brain Structures

One-way ANOVA demonstrated a significant effect of 1BnTIQ treatment (*F*[2,18] = 18.61, *P* < 0.01) on the level of DA in the striatum (Table 1). Duncan’s post hoc analysis demonstrated that both the acute and multiple administration of 1BnTIQ significantly decreased the level of DA (respectively, 40 and 25 %; *P* < 0.01). The statistical analysis indicated no effect of 1BnTIQ treatment (*F*[2,18] = 1.31, N.S.) on DOPAC concentration (Table 1). One-way ANOVA demonstrated a significant effect of 1BnTIQ treatment (*F*[2,18] = 5.81, *P* < 0.05) on the level of 3-MT in the striatum (Table 1). The post hoc analysis showed that the acute injection of 1BnTIQ reduced the concentration of 3-MT in the rat striatum (approximately 25 %, *P* < 0.05). The statistical analysis indicated a significant effect of 1BnTIQ treatment (*F*[2,18] = 4.81, *P* < 0.05) on the HVA concentration (Table 1). Duncan’s post hoc analysis demonstrated that the acute administration of 1BnTIQ significantly increased the level of HVA (approximately 60 %; *P* < 0.05).

**Table 1:** The comparison of the influence of acute and chronic administration of 1BnTIQ on dopamine (DA) metabolism in rat brain structures

Treatment	*N*	DA ng/g	DOPAC ng/g	3-MT ng/g	HVA ng/g
Striatum					
Saline	7	9894 ± 618	2158 ± 113	416 ± 30	1027 ± 90
1BnTIQ/25-1×	7	5817 ± 415**	1897 ± 83	316 ± 14**	1638 ± 171**
1BnTIQ/25-14×	7	7416 ± 356**^+^	1999 ± 142	388 ± 16^+^	1383 ± 146
*F*		*F*_(2/18)_ = 18.61 *P* < 0.00004	*F*_(2/18)_ = 1.31 NS	*F*_(2/18)_ = 5.81 *P* < 0.01	*F*_(2/18)_ = 4.81 *P* < 0.02
Nucleus accumbens					
Saline	7	9.089 ± 406	2.742 ± 98	320 ± 19	1.049 ± 69
1BnTIQ/25-1×	7	5.656 ± 505**	2.408 ± 180	195 ± 24**	1.245 ± 141*
1BnTIQ/25-14×	7	6.172 ± 358**	2.024 ± 94**^+^	233 ± 16**	926 ± 53^+^
*F*		*F*_(2/18)_ = 18.73 *P* < 0.00004	*F*_(2/18)_ = 7.64 *P* < 0.003	*F*_(2/18)_ = 10.26 *P* < 0.001	*F*_(2/18)_ = 4.81 *P* < 0.02

1BnTIQ (25 mg/kg i.p.) was administered acute or chronically during 14 consecutive days. Rats were decapitated 2 h after the injections. The results are expressed as the means ± SEM of seven samples (*n* = 7 animals per group). Data were analyzed with a one-way ANOVA followed by Duncan’s post hoc test. Statistical significance: * *P* < 0.05; ** *P* < 0.01 versus Saline group; ^+^ *P* < 0.05 versus acute 1BnTIQ group

One-way ANOVA indicated a significant effect of 1BnTIQ treatment (*F*[2,18] = 18.73, *P* < 0.01) on the level of DA in the nucleus accumbens (Table 1). The post hoc test showed that both the acute and multiple administration of 1BnTIQ significantly decreased the level of DA (respectively, 40 and 30 %; *P* < 0.01). Treatment with 1BnTIQ (*F*[2,18] = 7.64, *P* < 0.01) significantly affected the DOPAC concentration in the nucleus accumbens (Table 1). Duncan’s post hoc analysis demonstrated that the multiple administration of 1BnTIQ significantly decreased the level of DOPAC (approximately 25 %; *P* < 0.01). One-way ANOVA indicated a significant effect of 1BnTIQ treatment (*F*[2,18] = 10.26, *P* < 0.01) on the 3-MT concentration in the nucleus accumbens (Table 1). The post hoc test showed that both acute and multiple administration of 1BnTIQ significantly decreased the level of 3-MT (respectively 40 and 30 %; *P* < 0.01). The statistical analysis indicated a significant effect of 1BnTIQ treatment (*F*[2,18] = 4.81, *P* < 0.05) on the HVA concentration (Table 1). Duncan’s post hoc analysis demonstrated that the acute administration of 1BnTIQ significantly increased the level of HVA (approximately 20 %; *P* < 0.05).

##### The Effect of Acute 1MeTIQ Administration on Changes in Dopamine Metabolism Induced After the Chronic Administration of 1BnTIQ in Rat Brain Structures Table 2

#### Striatum

Two-way ANOVA indicated a significant effect of chronic treatment with 1BnTIQ (*F*[2,35] = 29.41, *P* < 0.01) and an acute dose of 1MeTIQ (*F*[2,35] = 8.88, *P* < 0.01) on the DA concentration in the striatum (Table 2). The interaction between 1BnTIQ and 1MeTIQ treatment was not significant (*F*[2,35] = 0.79, N.S.). Duncan’s post hoc analysis demonstrated that the multiple administration of 1BnTIQ significantly decreased the level of DA (approximately 25 %; *P* < 0.01). In contrast, 1MeTIQ co-administered with the last dose of 1BnTIQ completely antagonized the 1BnTIQ-induced reduction in DA (the DA level remained similar to the control level) (Table 2).

**Table 2:** The influence of acute administration of 1MeTIQ on changes induced by chronic treatment with 1BnTIQ in the concentration of dopamine (DA) and its metabolites

Treatment		*N*	DA (ng/g)	DOPAC (ng/g)	3-MT (ng/g)	HVA (ng/g)
Acute	Chronic					
Striatum						
Saline	Saline	7	9894 ± 618	2158 ± 113	416 ± 30	1027 ± 90
1MeTIQ/25	Saline	7	11055 ± 264	1432 ± 61**	620 ± 34**	288 ± 27**
1MeTIQ/50	Saline	7	10979 ± 502	1144 ± 47**	706 ± 38**	1049 ± 87
Saline	MeTIQ/25	6	7416 ± 356**	1999 ± 142	388 ± 16	1383 ± 146*
1MeTIQ/25	1BnTIQ/25	7	9649 ± 542^++^	1254 ± 64**^++^	587 ± 45**^++^	1072 ± 58^+^
1MeTIQ/25	1BnTIQ/25	7	9105 ± 118^++^	968 ± 79**^++^	703 ± 44**^++^	881 ± 60^++^
Effect of 1MeTIQ			*F*_(2/35)_ = 8.88 *P* < 0.0007	*F*_(2/35)_ = 64.73 *P* < 0.0000001	*F*_(2/35)_ = 36.49 *P* < 0.0000001	*F*_(2/35)_ = 18.87 *P* < 0.000003
Effect of 1BnTIQ			*F*_(2/35)_ = 29.41 *P* < 0.00004	*F*_(2/35)_ = 5.11 *P* < 0.03	*F*_(2/35)_ = 0.50 NS	*F*_(2/35)_ = 20.97 *P* < 0.00005
Interaction of 1MeTIQ + 1BnTIQ			*F*_(2/35)_ = 0.79 NS	*F*_(2/35)_ = 0.01 NS	*F*_(2/35)_ = 0.09 NS	*F*_(2/35)_ = 14.88 *P* < 0.00002
Nucleus accumbens						
Saline	Saline	7	9089 ± 406	2742 ± 98	320 ± 19	1049 ± 69
1MeTIQ/25	Saline	7	9796 ± 388	1742 ± 89**	386 ± 18	901 ± 71
1MeTIQ/50	Saline	7	9765 ± 1145	1406 ± 204**	402 ± 47	854 ± 129
Saline	1BnTIQ/25	6	6172 ± 358**	2024 ± 94**	233 ± 16*	926 ± 53
1MeTIQ/25	1BnTIQ/25	7	8316 ± 413^+^	1378 ± 85**^++^	373 ± 24^++^	693 ± 44**^+^
1MeTIQ/50	1BnTIQ/25	7	8351 ± 476^+^	1080 ± 95**^++^	467 ± 34**^++^	664 ± 56**^+^
Effect of 1MeTIQ			*F*_(2/35)_ = 4.40 *P* < 0.01	*F*_(2/35)_ = 54.39 *P* < 0.0000001	*F*_(2/35)_ = 17.01 *P* < 0.000007	*F*_(2/35)_ = 5.80 *P* < 0.006
Effect of 1BnTIQ			*F*_(2/35)_ = 17.97 *P* < 0.0001	*F*_(2/35)_ = 25.95 *P* < 0.00001	*F*_(2/35)_ = 0.27 NS	*F*_(2/35)_ = 8.75 *P* < 0.005
Interaction of 1MeTIQ + 1BnTIQ			*F*_(2/35)_ = 1.17 NS	*F*_(2/35)_ = 1.86 NS	*F*_(2/35)_ = 3.74 *P* < 0.03	*F*_(2/35)_ = 0.19 NS

1BnTIQ was chronically administered (25 mg/kg i.p.) during 14 consecutive days. In the mixed group, 1MeTIQ (50 mg/kg i.p.) was given once, 20 min before last 1BnTIQ administration. Rats were decapitated 2 h after the injections. The results are expressed as the means ± SEM of six to seven samples (*n* = 6–7 animals per group). Data were analyzed with a two-way ANOVA followed by Duncan’s post hoc test. Statistical significance: * *P* < 0.05; ** *P* < 0.01 versus Saline/Saline group; ^+^ *P* < 0.05, ^++^ *P* < 0.01 versus 1BnTIQ group

Two-way ANOVA demonstrated a significant effect of 1BnTIQ (*F*[2,35] = 5.11, *P* < 0.05) and 1MeTIQ (*F*[2,35] = 64.73, *P* < 0.01) on the DOPAC concentration in the striatum (Table 2). The interaction between 1BnTIQ and 1MeTIQ was not significant (*F*[2,35] = 0.01, N.S.). The post hoc analysis indicated that the acute administration of 1MeTIQ (at both doses examined) decreased the DOPAC concentration (approximately 30 and 45 %; *P* < 0.01) and that this effect was enhanced in the mixed groups (approximately 40 and 55 %) (Table 2).

The statistical analysis showed no effect of 1BnTIQ (*F*[2,35] = 0.5, N.S.) or the interaction between 1BnTIQ and 1MeTIQ (*F*[2,35] = 0.09; N.S.) on the levels of 3-MT in the rat striatum. In contrast, the effect of 1MeTIQ was significant (*F*[2,35] = 36.49, *P* < 0.01) (Table 2). Duncan’s post hoc test showed that both doses of 1MeTIQ administered alone increased the 3-MT concentration (respectively, 50 and 70 %, *P* < 0.01), and similar effects were observed in the mixed groups (Table 2).

Treatment with 1BnTIQ (*F*[2,35] = 20.97, *P* < 0.01) and 1MeTIQ (*F*[2,35] = 18.87, *P* < 0.01) significantly affected the HVA concentration in the striatum (Table 2). This analysis also revealed a significant interaction between 1BnTIQ and 1MeTIQ (*F*[2,35] = 14.88, *P* < 0.01). The post hoc analysis showed that the chronic administration of 1BnTIQ significantly increased the level of HVA (approx. 35 %, *P* < 0.05), and this effect was completely blocked through the administration of both concentrations of 1MeTIQ (Table 2).

**Fig. 2:**
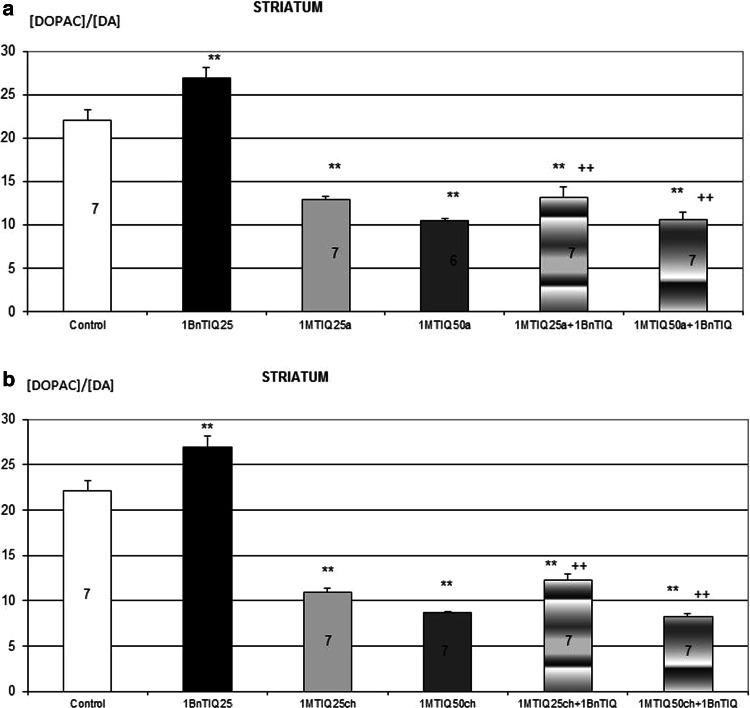
The influence of acute (**a**) and chronic (**b**) administration of 1MeTIQ on the elevation rate of dopamine oxidation induced by chronic treatment with 1BnTIQ. 1BnTIQ (25 mg/kg i.p.) was administered chronic during 14 consecutive days. 1MeTIQ (25 or 50 mg/kg i.p) was given acute (**a**) or chronic during 14 consecutive days (**b**). In the mixed group, 1MeTIQ was injected 20 min before 1BnTIQ administration. A control group was treated by saline. Rats were decapitated 2 h after the injections. The data are expressed as the means ± SEM (*n* = 6–7 animals). Data were analyzed with a two-way ANOVA, followed by Duncan’s post hoc test. Statistical significance: * *P* < 0.05; ** *P* < 0.01 versus saline-treated group; ^++^ *P* < 0.01 versus 1BnTIQ-treated group

Two-way ANOVA demonstrated that chronic treatment with 1BnTIQ significantly increased the rate of DA oxidation, measured as index [DOPAC]/[DA] (*P* < 0.01). This effect was completely inhibited through the acute administration of 1MeTIQ at both doses examined (Fig. 2a). Similarly, the rate of total DA catabolism (measured as index [HVA]/[DA]) was significantly (*P* < 0.01) increased after the multiple administration of 1BnTIQ, and this effect was antagonized with a single dose of 1MeTIQ (Fig. 2b).

#### Nucleus Accumbens

Two-way ANOVA indicated a significant effect of both 1BnTIQ (*F*[2,35] = 17.97, *P* < 0.01) and 1MeTIQ (*F*[2,35] = 4.4, *P* < 0.01) treatments on the DA concentration in the nucleus accumbens (Table 2). In addition, the interaction between 1BnTIQ and 1MeTIQ was not significant (*F*[2,35] = 1.17, N.S.). Duncan’s post hoc test showed that the chronic administration of 1BnTIQ decreased the DA concentration (approx. 30 %, *P* < 0.01). Both concentrations of 1MeTIQ partially antagonized this effect (Table 2).

The statistical analysis showed a significant effect of 1BnTIQ (*F*[2,35] = 25.95, *P* < 0.01) and 1MeTIQ (*F*[2,35] = 54.39, *P* < 0.01) treatments on the DOPAC levels. In contrast, the interaction between 1BnTIQ and 1MeTIQ (*F*[2,35] = 1.86, N.S.) was not significant (Table 2). The post hoc analysis demonstrated a reduction in the level of DOPAC through chronic treatment with 1BnTIQ (approximately 25 %), and both doses of 1MeTIQ (respectively, 40 and 50 %). In the mixed groups, this reduction was markedly enhanced (respectively, 50 and 60 %). (Table 2).

Two-way ANOVA further revealed no effect of the chronic administration of 1BnTIQ (*F*[2,35] = 0.27, N.S.) on the 3-MT concentration in the nucleus accumbens (Table 2). However, the effects of 1MeTIQ (*F*[2,35] = 17.01, *P* < 0.01) and the interaction between 1BnTIQ and 1MeTIQ were significant (*F*[2,35] = 3.74, *P* < 0.05). Duncan’s post hoc test showed that chronic treatment with 1BnTIQ (25 mg/kg) reduced the levels of 3-MT (approximately 30 %), and this effect was completely blocked using both doses of 1MeTIQ (Table 2).The statistical analysis indicated a significant effect of 1BnTIQ (*F*[2,35] = 8.75, *P* < 0.01) and 1MeTIQ (*F*[2,35] = 5.80, *P* < 0.01) treatments on the HVA levels, but the interaction between 1BnTIQ and 1MeTIQ (*F*[2,35] = 0.19, N.S.) was not significant. The post hoc analysis showed that treatment with 1BnTIQ concomitant with 1MeTIQ significantly reduced the HVA levels (approximately 35 %) (Table 2).

##### The Effect of Chronic 1MeTIQ Administration on Changes in Dopamine Metabolism Induced Through the Chronic Administration of 1BnTIQ in Rat Brain Structures Table 3

#### Striatum

Two-way ANOVA revealed a significant effect of chronic treatment with 1BnTIQ (*F*[2,36] = 41.37, *P* < 0.01) and the chronic administration of 1MeTIQ (*F*[2,36] = 10.18, *P* < 0.01) on the DA concentration in the striatum (Table 3). There was no significant interaction between the chronic administration of 1BnTIQ and multiple injections with 1MeTIQ (*F*[2,36] = 2.01, N.S.). Duncan’s post hoc test showed that the chronic administration of 1BnTIQ reduced the DA concentration (approximately 25 %; *P* < 0.01), while the multiple administration of 1MeTIQ (in both doses) increased neurotransmitter (by 20 %; *P* < 0.05). Additionally, when the chronic administration of 1BnTIQ (25 mg/kg) was coupled with the chronic administration of 1MeTIQ, the concentration of dopamine returned to the control level (Table 3).

**Table 3:** The influence of chronic administration of 1MeTIQ on changes induced by chronic treatment with 1BnTIQ in the concentration of dopamine (DA) and its metabolites

Treatment		*N*	DA (ng/g)	DOPAC (ng/g)	3-MT (ng/g)	HVA (ng/g)
Chronic	Chronic					
Striatum						
Saline	Saline	7	9894 ± 618	2158 ± 113	416 ± 30	1027 ± 90
1MeTIQ/25	Saline	7	11441 ± 254*	1255 ± 59**	603 ± 17**	930 ± 47
1MeTIQ/50	Saline	7	11259 ± 579*	976 ± 55**	737 ± 35**	868 ± 48
Saline	1BnTIQ/25	7	7416 ± 356**	1999 ± 142	388 ± 16	1383 ± 146**
1MeTIQ/25	1BnTIQ/25	7	8390 ± 239*	1029 ± 49**^++^	616 ± 19**^++^	853 ± 59^++^
1MeTIQ/50	1BnTIQ/25	7	9924 ± 416^++^	823 ± 51**^++^	705 ± 37**^++^	807 ± 53^++^
Effect of 1MeTIQ			*F*_(2/36)_ = 10.18 *P* < 0.0003	*F*_(2/36)_ = 104.27 *P* < 0.0000001	*F*_(2/36)_ = 70.60 *P* < 0.0000001	*F*_(2/36)_ = 11.65 *P* < 0.0001
Effect of 1BnTIQ			*F*_(2/36)_ = 41.37 *P* < 0.0000001	*F*_(2/36)_ = 6.49 *P* < 0.01	*F*_(2/36)_ = 0.48 NS	*F*_(2/36)_ = 1.18 NS
Interaction of 1MeTIQ + 1BnTIQ			*F*_(2/36)_ = 2.01 NS	*F*_(2/36)_ = 0.108 NS	*F*_(2/36)_ = 0.41 NS	*F*_(2/36)_ = 4.47 *P* < 0.01
Nucleus accumbens						
Saline	Saline	7	9089 ± 406	2742 ± 98	320 ± 19	1049 ± 69
1MeTIQ/25	Saline	7	9552 ± 669	1295 ± 105**	354 ± 25	689 ± 79**
1MeTIQ/50	Saline	7	8892 ± 363	1050 ± 60**	405 ± 29*	593 ± 33**
Saline	1BnTIQ/25	7	6172 ± 358**	2024 ± 94**	233 ± 16*	926 ± 53
1MeTIQ/25	1BnTIQ/25	7	6971 ± 373**^++^	1038 ± 74**^++^	369 ± 22^++^	518 ± 43**^++^
1MeTIQ/50	1BnTIQ/25	7	8442 ± 581^++^	871 ± 77**^++^	393 ± 28*^++^	509 ± 24**^++^
Effect of 1MeTIQ			*F*_(2/36)_ = 2.43 NS	*F*_(2/36)_ = 159.28 *P* < 0.0000001	*F*_(2/36)_ = 14.17 *P* < 0.00002	*F*_(2/36)_ = 39.26 *P* < 0.0000001
Effect of 1BnTIQ			*F*_(2/36)_ = 26.21 *P* < 0.00001	*F*_(2/36)_ = 29.92 *P* < 0.000004	*F*_(2/36)_ = 2.05 NS	*F*_(2/36)_ = 8.25 *P* < 0.006
Interaction of 1MeTIQ + 1BnTIQ			*F*_(2/36)_ = 3.98 *P* < 0.02	*F*_(2/36)_ = 5.74 *P* < 0.006	*F*_(2/36)_ = 2.48 NS	*F*_(2/36)_ = 0.33 NS

1BnTIQ was chronically administered (25 mg/kg i.p.) during 14 consecutive days. In the mixed group, 1MeTIQ (50 mg/kg i.p.) was given also chronically, 20 min before each 1BnTIQ administration. Rats were decapitated 2 h after the injections. The results are expressed as the means ± SEM of seven samples (*n* = 7 animals per group). Data were analyzed with a two-way ANOVA followed by Duncan’s post hoc test. Statistical significance: * *P* < 0.05; ** *P* < 0.01 versus Saline/Saline group; ^+^ *P* < 0.05; ^++^ *P* < 0.01 versus 1BnTIQ group

Two-way ANOVA demonstrated a significant effect of chronic treatment with 1BnTIQ (*F*[2,36] = 6.49, *P* < 0.01) and the multiple administration of 1MeTIQ (*F*[2,36] = 104.27, *P* < 0.01) on the DOPAC concentration in the striatum (Table 3). Notably, the interaction between 1BnTIQ and 1MeTIQ was not significant (*F*[2,36] = 0.11, N.S.). The post hoc analysis showed that chronic 1MeTIQ administration strongly decreased the DOPAC concentration (respectively, 40 and 55 %; *P* < 0.01); this effect was enhanced in the mixed group (respectively, 55 and 65 %; *P* < 0.01) (Table 3).

The statistical analysis revealed no effect of chronic treatment with 1BnTIQ (*F*[2,36] = 0.48, N.S.) on the level of 3-MT (Table 3). Chronic treatment with 1MeTIQ (*F*[2,36] = 70.6, *P* < 0.01) significantly affected the 3-MT concentration. There was no interaction between the chronic administration of 1BnTIQ and the multiple administration of 1MeTIQ (*F*[2,36] = 0.41, N.S.). Duncan’s post hoc test showed that chronic treatment with 1MeTIQ (in both doses) increased the 3-MT concentration (approximately 145 and 180 %; *P* < 0.01). A similar effect was observed with the chronic administration of 1BnTIQ (25 mg/kg) coupled with the chronic administration of 1MeTIQ, (Table 3).

Two-way ANOVA demonstrated no effect of chronic 1BnTIQ treatment (*F*[2,36] = 1.18, N.S.) on the HVA concentration in the striatum (Table 3). In contrast, the chronic administration of 1MeTIQ(*F*[2,36] = 11.65, *P* < 0.01) significantly affected the level of HVA. An interaction between 1BnTIQ and 1MeTIQ was also detected (*F*[2,36] = 4.47, *P* < 0.01). The post hoc analysis showed that 1BnTIQ increased the HVA concentration (approximately 135 %; *P* < 0.01); this effect was completely blocked through chronic treatment with both concentrations of 1MeTIQ (Table 3).

**Fig. 3:**
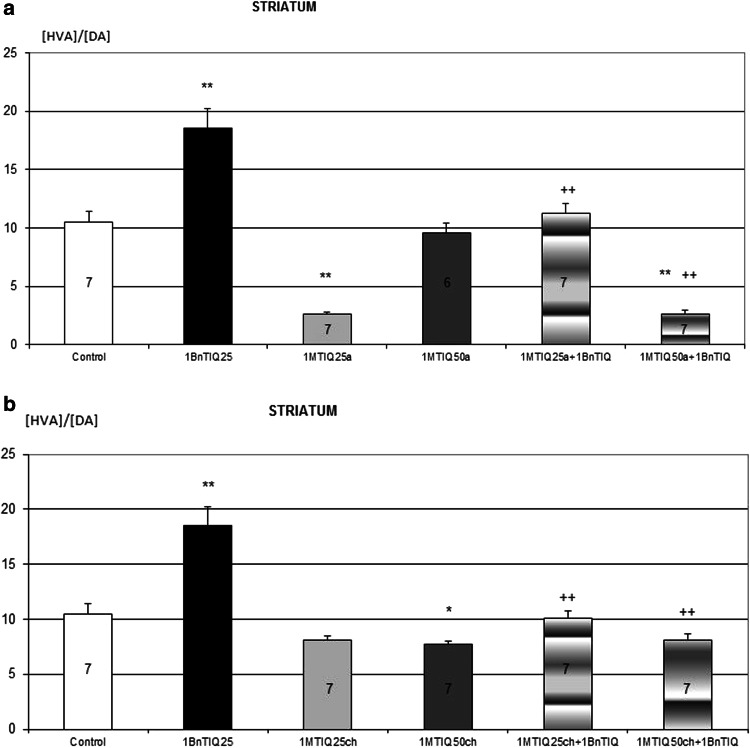
The influence of acute (**a**) and chronic (**b**) administration of 1MeTIQ on the elevation rate of dopamine catabolism induced by chronic treatment with 1BnTIQ. 1BnTIQ (25 mg/kg i.p.) was administered chronic during 14 consecutive days. 1MeTIQ (25 or 50 mg/kg i.p) was given acute (**a**) or chronic during 14 consecutive days (**b**). In the mixed group, 1MeTIQ was injected 20 min before 1BnTIQ administration. A control group was treated by saline. Rats were decapitated 2 h after the injections. The data are expressed as the means ± SEM (*n* = 6–7 animals). Data were analyzed with a two-way ANOVA, followed by Duncan’s post hoc test. Statistical significance: * *P* < 0.05; ** *P* < 0.01 versus saline-treated group; ^++^ *P* < 0.01 versus 1BnTIQ-treated group

Two-way ANOVA indicated that repeated treatment with 1BnTIQ significantly increased the rate of DA oxidation measured as index [DOPAC]/[DA] (*P* < 0.01). This effect was completely inhibited through the chronic administration of 1MeTIQ at both doses examined (Fig. 3a). Similarly, the rate of total DA catabolism (measured as index [HVA]/[DA]) was significantly (*P* < 0.01) increased through the multiple administration of 1BnTIQ, and this effect was antagonized through the chronic administration of 1MeTIQ (Fig. 3b).

#### Nucleus Accumbens

Two-way ANOVA revealed a significant effect of chronic 1BnTIQ treatment (*F*[2,36] = 26.21, *P* < 0.01) on the DA concentration in the nucleus accumbens (Table 3). In contrast, multiple 1MeTIQ administration produced no effect on the level of DA (*F*[2,36] = 2.43, N.S.). The statistical analysis revealed a significant interaction between the chronic administration of 1BnTIQ and 1MeTIQ (*F*[2,36] = 3.98, *P* < 0.05). The post hoc test indicated that the multiple injection of 1BnTIQ decreased the level of DA (approximately 30 %, *P* < 0.01) and that this effect was antagonized after chronic treatment with 1MeTIQ (Table 3).

Two-way ANOVA demonstrated a significant effect of chronic treatment with 1BnTIQ (*F*[2,36] = 29.92, *P* < 0.01) and the multiple administration of 1MeTIQ (*F*[2,36] = 159.28, *P* < 0.01) on the DOPAC concentration in rat nucleus accumbens (Table 3). An interaction between 1BnTIQ and l-DOPA was also detected (*F*[2,36] = 5.74, *P* < 0.01). The post hoc analysis showed that chronic injections of both 1BnTIQ and 1MeTIQ reduced the DOPAC concentration (respectively, 25, 55 and 60 %; *P* < 0.01); this effect was intensified in the mixed groups (respectively, 65 and 70 %) (Table 3).

Two-way ANOVA revealed no effect of the chronic administration of 1BnTIQ (*F*[2,36] = 2.05, N.S.) on the level of 3-MT in the nucleus accumbens. The same statistical analysis indicated that chronic treatment with 1MeTIQ (*F*[2,36] = 14.17, *P* < 0.01) significantly affected the 3-MT concentration (Table 3). The statistical analysis revealed no interaction between 1BnTIQ and 1MeTIQ (*F*[2,36] = 2.48, N.S.). Duncan’s post hoc test showed that multiple treatment with 1BnTIQ decreased the level of 3-MT (approximately 30 %; *P* < 0.05), and this effect was reversed through chronic treatment at both concentrations of 1MeTIQ (Table 3).

Two-way ANOVA revealed a significant effect of chronic treatment with both 1BnTIQ (*F*[2,36] = 8.25, *P* < 0.01) and 1MeTIQ (*F*[2,36] = 39.26, *P* < 0.01) on the HVA concentration in the nucleus accumbens (Table 3). The interaction between the chronic administration of 1BnTIQ and chronic treatment with 1MeTIQ was not significant (*F*[2,36] = 0.33, N.S.). The post hoc analysis demonstrated that chronic treatment with both doses of 1MeTIQ strongly decreased the HVA concentration (respectively, 30 and 40 %; *P* < 0.01); this effect was intensified in the mixed groups (approximately 50 %) (Table 3).

##### The Effect of Chronic 1MeTIQ Administration on 1BnTIQ-Produced Changes in the Concentration of EAA in the Rat Frontal Cortex (Table 4)

One-way ANOVA demonstrated a significant effect of treatment (*F*[6,35] = 4.77, *P* < 0.01) on the concentration of aspartate in the frontal cortex (Table 4). Duncan’s post hoc test showed that the chronic administration of 1BnTIQ increased the aspartate concentration (approximately 125 %; *P* < 0.01) and that this effect was completely inhibited through the chronic administration of 1MeTIQ (*P* < 0.01). The statistical analysis revealed no effect of treatment (*F*[6,35] = 1.65, N.S.) on the level of glutamate in the frontal cortex (Table 4). However, post hoc analysis indicated that the chronic administration of 1BnTIQ increased the concentration of glutamate (*P* < 0.05) and that this effect was blocked through the multiple administration of 1MeTIQ (25 mg/kg i.p.) (Table 4).

**Table 4:** The influence of chronic treatment with 1MeTIQ and 1BnTIQ on the concentration of aspartate (ASP) and glutamate (GLU) in rat front al cortex

Treatment	*N*	ASP (ng/20 µl)	GLU (ng/20 µl)
Frontal cortex			
Saline	5	464 ± 15	1998 ± 53
1BnTIQ/25 × 1	7	447 ± 17	2005 ± 42
1BnTIQ/25 × 14	6	588 ± 42**	2273 ± 161*
1MeTIQ/25 × 14	5	498 ± 10	2133 ± 100
1MeTIQ/50 × 14	6	520 ± 21	1988 ± 38
1MeTIQ/25 × 14 + 1BnTIQ/25 × 14	6	477 ± 16^++^	1998 ± 55^+^
1MeTIQ/50 × 14 + 1BnTIQ/25 × 14	6	530 ± 15	2067 ± 45
* F*		*F*_(6/35)_ = 4.77 *P* < 0.01	*F*_(6/35)_ = 1.65 N.S.

1BnTIQ (25 mg/kg i.p.) was administered acute or chronically during 14 consecutive days. 1MeTIQ was given in two doses (25 and 50 mg/kg i.p.) chronically. In the mixed group, 1MeTIQ was given also chronically, 20 min before each 1BnTIQ administration. Rats were decapitated 2 h after the injections. The results are expressed as the means ± SEM of five to seven samples (*n* = 5–7 animals per group). Data were analyzed with a one-way ANOVA followed by Duncan’s post hoc test. Statistical significance: * *P* < 0.05; ** *P* < 0.01 versus saline group; ^+^ *P* < 0.05; ^++^ *P* < 0.01 versus 1BnTIQ group

### Immunoblotting

**Fig. 4:**
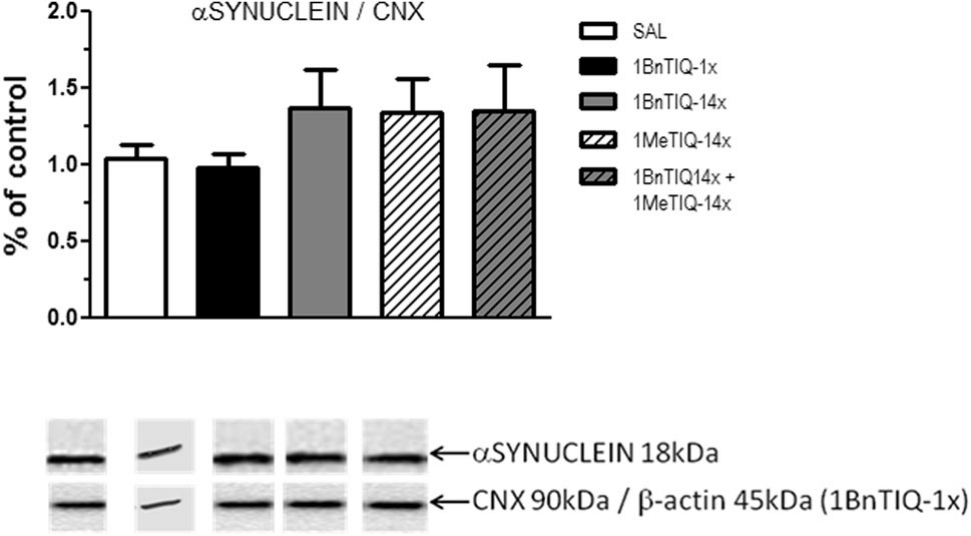
The influence of chronic 1MeTIQ administration on 1BnTIQ-induced changes in α-synuclein level. 1BnTIQ was administered (25 mg/kg i.p.) acute or chronically for 14 consecutive days. In the mixed group, 1MeTIQ (50 mg/kg i.p.) was administered chronically, 20 min before 1BnTIQ administration. Control rats were treated with the appropriate vehicle. Rats were killed by decapitation 2 h after last drug injections and substantia nigra was dissected for analysis. The data are expressed as the means ± SEM. (*n* = 6–7 animals). Data were analyzed with a one-way ANOVA, followed by Duncan’s post hoc test. Statistical significance: * *P* < 0.05; ** *P* < 0.01 versus saline-treated group; ^+^ *P* < 0.05 versus 1BnTIQ-treated group

Figure 4 shows that the acute administration of 1BnTIQ (25 mg/kg i.p.) did not change the level of α-synuclein in the substantia nigra, whereas the multiple administration of 1BnTIQ weakly (not significant) elevated the α-synuclein concentration. A similar effect (non-significant increase in the level of α-synuclein) was observed after the chronic administration of 1MeTIQ (50 mg/kg i.p.) and in the combined group (Fig. 4).

**Fig. 5:**
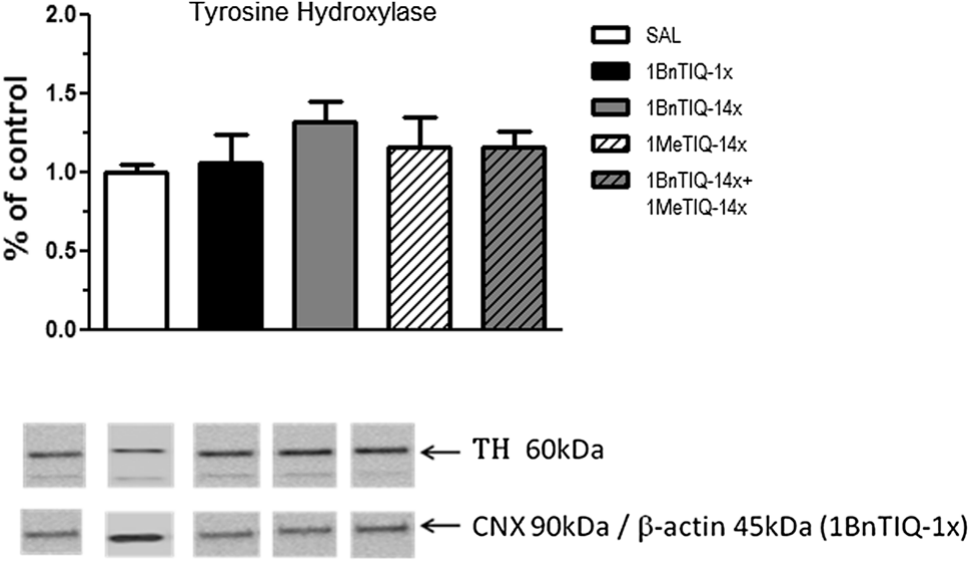
The influence of chronic 1MeTIQ administration on 1BnTIQ-induced changes in tyrosine hydroxylase concentration. 1BnTIQ was administered (25 mg/kg i.p.) acute or chronically for 14 consecutive days. In the mixed group, 1MeTIQ (50 mg/kg i.p.) was administered chronically, 20 min before 1BnTIQ administration. Control rats were treated with the appropriate vehicle. Rats were killed by decapitation 2 h after last drug injections and substantia nigra was dissected for analysis. The data are expressed as the means ± SEM. (*n* = 6–7 animals). Data were analyzed with a one-way ANOVA, followed by Duncan’s post- hoc test. Statistical significance: * *P* < 0.05; ** *P* < 0.01 versus saline-treated group; ^*+*^ *P* < 0.05 versus 1BnTIQ-treated group

As demonstrated in Fig. 5, neither acute nor chronic treatment with 1BnTIQ (25 mg/kg i.p.) changed the concentration of tyrosine hydroxylase. In addition, treatment with 1MeTIQ (50 mg/kg i.p.) alone and concomitant with 1BnTIQ did not influence the level of tyrosine hydroxylase (Fig. 5).

## Discussion

The chronic administration of 1BnTIQ induced parkinsonism in primates, and this effect might reflect idiopathic Parkinson’s disease (PD) (Abe et al. 2005). The neurotoxin 1BnTIQ causes parkinsonism in humans, and this molecule is present in the cerebrospinal fluid of parkinsonian patients at approximately three times the concentration detected in healthy individuals (Kotake et al. 1995). These data clearly demonstrated that chronic treatment with a low dose of 1BnTIQ could represent a good animal model of PD. Distinct disturbances in the function of dopamine neurons generated through the repeated administration of 1BnTIQ might imitate the progressive character of PD. The results obtained in the present study confirm those of earlier studies and show that both the acute and chronic administration of 1BnTIQ significantly disturbed dopamine (DA) metabolism in the rat brain (Wąsik et al. 2009). Interestingly, acute treatment with 1BnTIQ produced a stronger effect on DA metabolism than multiple administration, which suggests the development of tolerance after the chronic administration of 1BnTIQ (Table 1). However, both the single and repeated administration of 1BnTIQ significantly reduced the DA concentration in the brain structures, associated with the DA-mediated oxidative stress, as observed in the surviving dopaminergic terminals of PD patients (Antkiewicz-Michaluk et al. 2001). Previous studies have shown that 1BnTIQ strongly potentiates MAO-dependent DA oxidation, thereby increasing free radical production and damage to DA cells (Wąsik et al. 2009). However, 1BnTIQ inhibits COMT activity and significantly decreases the concentration of 3-MT, an extraneuronal metabolite of DA, with a different neuroprotective effect (Miller et al. 1996). We also present the first investigation of the influence of the chronic administration of 1BnTIQ on the level of the excitatory amino acids (EAA), aspartate (ASP) and glutamate (GLU), in rat frontal cortex.

Using this animal model of PD, we investigated the effect of the neuroprotective endogenous amine, 1MeTIQ. As an endogenous inhibitor of MAO activity, 1MeTIQ shifts DA catabolism toward COMT-dependent *O*-methylation, thereby increasing the concentration of the extraneuronal DA metabolite, 3-MT (Antkiewicz-Michaluk et al. 2001; Patsenka and Antkiewicz-Michaluk 2004). Indeed, we previously observed that 1MeTIQ possesses neuroprotective activity in different animal models of neurodegeneration and protects neurons against glutamate- and kainite-induced excitotoxicity (Antkiewicz-Michaluk et al. 2006, 2014).

In the present study, we demonstrated that both the acute and repeated administration of 1MeTIQ completely reversed the toxic effects of 1BnTIQ on DA neurons. Additionally, the chronic administration of 1MeTIQ antagonized the 1BnTIQ-induced increase of the EAA concentration in the rat frontal cortex (Table 4). These data suggest that 1MeTIQ might participate in the EAA transmission; however, in the present study, we investigated the entire pool of GLU localized in the tissue. The chronic administration of 1BnTIQ increased the total level of EAA, potentially leading to excitotoxicity. Psychostimulants, such as nicotine, also increase the concentration of GLU in the prefrontal cortex, which increases neuronal activity (Falasca et al. 2014; Shameem and Patel 2012). The data from other studies have suggested that the systemic administration of the well-known neurotoxin 1-methyl-1-phenyl-1,2,3,6-tetrahydropyridine (MPTP) in monkeys increases glutamatergic neurotransmission as an essential step in the evolution of parkinsonian symptoms (Bergman et al. 1990; Mitchell et al. 1989). The glutamate antagonist kynurenate reverses akinesia in MPTP-treated marmosets following focal injection into the medial pallidum (Brotchie et al. 1991). These results indicate that the selective reduction of glutamatergic overactivity might be an effective strategy for the treatment of PD.

The behavioral test showed that chronic treatment with 1BnTIQ significantly elevated the locomotor activity in rats (Fig. 1). Interestingly, an acute dose of 1BnTIQ (50 mg/kg i.p.) produced the opposite effect, showing the reduction of exploratory activity in rats, as previously demonstrated (Wąsik et al. 2009). The hyperactivity of rats observed after the multiple administration of 1BnTIQ might reflect the 1BnTIQ-induced impairment of DA storage, and this reserpine-like inhibition of the function of vesicular monoamine transporter (VMAT) has been previously demonstrated in an in vivo microdialysis study (Wąsik et al. 2014). Importantly, both the acute and chronic administration of 1MeTIQ (50 mg/kg i.p.) completely antagonized 1BnTIQ-induced hyperactivity (Fig. 1a, b) and reduced exploratory locomotor activity below that in the control group. Notably, the development of tolerance after the repeated administration of 1MeTIQ was not observed. This effect of 1MeTIQ can be explained by two mechanisms: (1) As a partial dopamine agonist, 1MeTIQ could block the active conformation of the DA receptor (Antkiewicz-Michaluk et al. 2014); and (2) 1MeTIQ shifts DA catabolism toward COMT-dependent *O*-methylation pathway, thereby significantly increasing the extraneuronal metabolite concentration of 3-MT (Antkiewicz-Michaluk et al. 2001). Moreover, 3-MT shows affinity to the α-adrenergic receptor as an antagonist, and this molecule might play an important physiological role as an inhibitory regulator, counteracting the excessive stimulation of catecholaminergic neurons (Antkiewicz-Michaluk et al. 2008).

Biochemical studies have shown that the acute and chronic administration of 1BnTIQ markedly decreased the dopamine level in the extrapyramidal (striatum) and mesolimbic (nucleus accumbens) dopamine structures in the rat brain (Table 1). Post-mortem clinical trials demonstrated a total degradation of nigrostriatal pathway, but also other dopaminergic and noradrenergic structures (Tong et al. 2006).The toxic effect of 1BnTIQ was completely blocked through the acute and chronic administration of 1MeTIQ (25 and 50 mg/kg) in both investigated structures: striatum and nucleus accumbens (Tables 2, 3). The simultaneous administration of 1MeTIQ with 1BnTIQ not only restored the DA concentration to the control level but also decreased the DOPAC concentration, thereby inhibiting DA oxidation (Tables 2, 3). Antagonism of 1MeTIQ compared to changes induced by 1BnTIQ is better marked in the striatum (Tables 2, 3). We also demonstrated (Figs. 2, 3) that 1BnTIQ significantly elevated both the rate of the final DA catabolism, calculated as the ratio [HVA]/[DA], and DA oxidation, calculated as the index [DOPAC]/[DA]. Both the acute and chronic administration of 1MeTIQ completely antagonized the effect of 1BnTIQ on the rate of DA catabolism (Figs. 2, 3). The biochemical effects of 1MeTIQ might reflect the restrictive influence of this molecule on MAO activity. It has been previously demonstrated that 1MeTIQ acts as a reversible inhibitor of MAO-A and MAO-B activity (Patsenka and Antkiewicz-Michaluk 2004; Patsenka and Michaluk 2004; Antkiewicz-Michaluk et al. 2006). Interesting is that l-deprenyl (which similarly to 1MeTIQ is an MAO inhibitor) decreases the endogenous 1BnTIQ content in the mouse brain (Kotake et al. 1998).

Additionally, we showed that the chronic administration of 1BnTIQ increased the concentration of both ASP and GLU in the rat frontal cortex (Table 4). In this case, the chronic administration of 1MeTIQ at a lower dose (25 mg/kg) antagonized this effect. These results are consistent with previous in vitro experiments, demonstrating the inhibition through 1MeTIQ glutamate-induced excitotoxicity, particularly that mediated through NMDA receptors, and this effect might be an important factor in the mechanism of neuroprotection through 1MeTIQ (Antkiewicz-Michaluk et al. 2006).

The potential neuroprotective activity of 1MeTIQ is of obvious interest because this compound is present in mammalian brains (Yamakawa and Ohta 1997, 1999; Yamakawa et al. 1999). These data are consistent with in vitro studies reporting that 1MeTIQ prevents the neurotoxic action of the four dopaminergic neurotoxins, MPTP, 6-OHDA, rotenone, and 1BnTIQ, in cultured mesencephalic neurons (Kotake et al. 2005). However, in the present study, acute and chronic treatment with 1BnTIQ did not change the levels of alpha-synuclein protein in the substantia nigra (Fig. 4). Moreover, the data obtained in the present study showed no effect of the repeated administration of 1BnTIQ on the tyrosine hydroxylase (TH) concentration in the substantia nigra (Fig. 5). Similar effects were obtained by Abe et al. (2001a), they reported that administration of 1BnTIQ did not reduce TH-positive cells in the substantia nigra in C57BL mouse. Notably, in vitro studies presented different results from in vivo studies. Shavali et al. (2004) demonstrated that 1BnTIQ increased α-synuclein expression and caused nuclear damage in human dopaminergic cells. Indeed, we should consider that in vivo experiments are not always consistent with in vitro research using cell culture. In vivo experiments often demonstrate compensatory mechanisms that might mask the toxic effects of the substance used. However, the other authors used TH immunohistochemistry to establish in vivo a potential protective effect of 1MeTIQ in the case when the death of dopaminergic cells was induced by MPP+ and TIQ (Abe et al. 2001b; Parrado et al. 2000). These papers confirmed the neuroprotective activity of 1MeTIQ, and demonstrated a significant antagonism of 1MeTIQ against neurotoxin-induced loss of TH-positive cells in the substantia nigra.

In conclusion, the results obtained in the present study clearly confirm that 1MeTIQ possesses essential neuroprotective properties in an animal model of Parkinson`s disease. Additionally, the neuroprotective effect of 1MeTIQ did not induce the development of tolerance after chronic administration and might restore the function of dopamine neurons, even after partial damage, which suggests their clinical relevance.
